# Multidrug resistance, biofilm formation, and virulence genes of *Escherichia coli* from backyard poultry farms

**DOI:** 10.14202/vetworld.2021.2869-2877

**Published:** 2021-11-10

**Authors:** Theeb Al-Marri, Abdulla Al-Marri, Reham Al-Zanbaqi, Ahmad Al Ajmi, Mahmoud Fayez

**Affiliations:** 1 Al Ahsa Veterinary Diagnostic Laboratory, Ministry of Environment, Water and Agriculture, Al-Ahsa 31982, Saudi Arabia; 2 Veterinary Diagnostic Laboratory, Department of Animal Resources, Doha, Qatar; 3 Veterinary Diagnostic Laboratory , Ministry of Environment, Water and Agriculture, Riyadh, Saudi Arabia; 4 The Central Laboratories for Veterinary, Agriculture, and Fisheries, East Amghara 21422, State of Kuwait, Kuwait; 5 Department of Bacteriology, Veterinary Serum and Vaccine Research Institute, Ministry of Agriculture, Cairo 11381, Egypt

**Keywords:** backyard broilers, *Escherichia coli*, multidrug resistance, virulence genes

## Abstract

**Background and Aim:**

Backyard chicken flocks have traditionally been regarded as an essential food source in developed countries; however, they may act as reservoirs and spread various zoonotic bacterial pathogens. This study was designed to investigate the prevalence, phenotypic resistance, biofilm formation (BF), and pathotypes of *Escherichia coli* isolates from backyard poultry farms.

**Materials and Methods:**

Cloacal swabs (n=150) and internal organs (n=150) were collected from 30 backyard chicken flocks; 20 of them were experiencing systemic infection, and the other ten were apparently healthy. Samples were bacteriologically examined for *E. coli* isolation. Isolates were identified biochemically by the VITEK^®^ 2 COMPACT system (BioMérieux, France). For molecular identification, *16S rRNA* was amplified and sequenced. Ten antimicrobials were selected for *E. coli* antimicrobial susceptibility testing. The minimum inhibitory concentration for each antimicrobial was determined. The extended-spectrum β-lactamase activity in isolates was investigated using cephalosporin/clavulanate combination disks. The ability of isolates for BF was determined by the microtiter plate method. Thirteen virulence genes linked to different *E. coli* pathotypes and two serotype-related genes were investigated by real-time polymerase chain reaction.

**Results:**

Eighty-six *E. coli* strains were isolated from 30 backyard chicken flocks. The isolates were biochemically identified to the species level. Genetically, sequences of the *16S rRN*A gene showed >98% identity with *E. coli* in the National Center for Biological Information database. The frequency of isolation from diseased flocks was significantly higher (p<0.05) than apparently healthy flocks; 63.9% of the isolates were recovered from cloacal swabs and 36.04% were recovered from internal organs. *E. coli* isolates showed high resistance to ampicillin (AMP; 75.6%), gentamicin (39.5%), and tetracycline (29.1%). However, none of the isolates were resistant to imipenem. A variable drug resistance profile for *E. coli* isolates was reported. Twenty-one (24.4%) isolates were sensitive to all ten antimicrobials. Seven (8.1%) isolates were resistant only to AMP, and 28 (32.6%) were resistant to two antimicrobials, whereas the remaining 30 (34.9%) isolates showed multidrug resistance (MDR). Of the 86 isolates, 8 (9.3%) were confirmed as extended-spectrum β-lactamase (ESBL)-producing *E. coli* by the combination disk diffusion method. All ESBL isolates were MDR with an MDR index of 0.5-0.6. Fifty-seven (66.3%) isolates were capable of forming biofilms; 22 (25.6%) of them were strong biofilm producers, 24 (27.9%) moderate producers, and 11 (12.8%) weak producers. A statistically significant pairwise correlation was obtained for MDR versus BF (r=0.512) and MDR index versus BF (r=0.556). Based on virulence gene profiles, five pathotypes were identified, including enteropathogenic *E. coli* (39.5%), avian pathogenic *E. coli* (32.53%), enterohemorrhagic *E. coli* (EHEC; 9.3%), enterotoxigenic *E. coli* (ETEC; 5.8%), and enteroaggregative *E. coli* (EAEC; 1.2%). The lower frequency of EAEC and ETEC was statistically significant than other pathotypes. Three isolates were identified as O157 based on the detection of the *rbfO157* gene.

**Conclusion:**

This study reported a high prevalence of MDR, suggesting the misuse of antimicrobials in backyard chicken farms. The emergence of ESBL and EHEC isolates in backyard chickens is a public health concern. Furthermore, the backyard flocks environment may harbor different pathogenic bacteria that may enhance the persistence of infection and the transmission to in-contact humans. Regular monitoring for the occurrence of MDR and the zoonotic pathotypes among *E. coli* in backyard chicken flocks is recommended, as these bacteria can transmit to humans through food products or contaminated environments.

## Introduction

*Escherichia coli* is a facultative anaerobic Gram-negative bacterium of the family Enterobacteriaceae. It is ubiquitous in the intestine of humans, animals, and birds as part of the intestinal flora. However, several strains that have acquired specific virulence genes have been incriminated in intestinal and extraintestinal infections [1]. Based on the combination of virulence genes, pathogenic *E. coli* are grouped into six pathotypes: Enterohemorrhagic *E. coli* (EHEC), enteropathogenic *E. coli* (EPEC), enteroinvasive *E. coli*, enterotoxigenic *E. coli* (ETEC), and diffusely adherent *E. coli* [2]. *E. coli* pathotypes incriminated in extraintestinal infections have been named Extraintestinal Pathogenic *E. coli* [3]. In poultry, avian pathogenic *E. coli* (APEC) is responsible for extraintestinal infections, causing colibacillosis [4].

EHEC strains encode the Shiga toxin gene and elaborate the Shiga toxin, causing life-threatening problems in humans following systemic absorption. They have been associated with hemolytic uremic syndrome and hemorrhagic colitis in humans, requiring hospitalization and extensive care, with significant mortality in children and the elderly [5]. ETEC binds to small intestinal enterocytes and secretes heat-stable and/or heat-labile enterotoxins, causing watery diarrhea. EPEC also binds to small intestinal enterocytes and destroys the normal microvillar architecture, resulting in inflammatory changes and diarrhea [6].

Animals and birds have been reported as reservoirs for pathogenic *E. coli*, which can spread between them and other livestock. Moreover, feces from reservoirs contaminate the environment; hence, humans become at risk of infection through direct contact with carrier animals or consumption of contaminated water and food [5].

Antibiotic resistance has been identified as a global public health issue. International health organizations have now elevated antibiotic resistance to one of the top health concerns of the 21^st^ century. *E. coli* has been used to monitor antimicrobial resistance in food animals because it is ubiquitous in the intestine. Moreover, some *E. coli* isolates carried by poultry are recognized as a potential source of antimicrobial resistance genes that may transmit to humans [7,8].

Biofilms are defined as the matrix that encloses bacterial populations that have aggregated to each other and adhered to surfaces and/or interfaces [9]. Biofilms are a significant public health issue because of their association with bacterial resistance to antimicrobials. Biofilm-associated bacteria can be up to 1000 times more resistant to antimicrobial therapy than their counterparts in the planktonic phase [10]. Regarding *E. coli*, biofilm formation (BF) contributes to the occurrence of different infections and makes their eradication difficult. The prevalence, mechanism of formation, and medical impact of biofilm in *E. coli* from humans, animals, and birds have been reviewed [11,12].

In Saudi Arabia, *E. coli* resistance profiles and pathotypes have been identified in human, animal, and food samples [13-17]. However, to the best of the authors’ knowledge, no accessible literature has documented *E. coli* pathotypes and phenotypes from backyard broilers in Saudi Arabia. Accordingly, this study aimed to investigate the prevalence, phenotypes, BF, and pathotypes of *E. coli* strains in backyard broilers.

## Materials and Methods

### Ethical approval

The study was approved by the Animal Ethics protocols established by the National Committee of Bio-Ethics, King Abdul-Aziz City of Science and Technology, and Royal Decree No. M/59.

### Study period and location

The study was conducted from November 2019 to December 2020. The study was carried out in Al-Hofuf city, in the eastern region, Saudi Arabia (N25 22 44.12, E49 35 12.51).

### Samples

Cloacal swabs and internal organs (liver, heart, blood, and air sacs) were collected from backyard broiler flocks (n=20) experiencing systemic infections (deaths with or without respiratory signs and diarrhea).

Five live diseased birds from each flock were autopsied, and internal organs were aseptically collected in a sterile screw-capped container. Cloacal swabs were collected by vigorous swabbing of the mucosal wall. In addition, control non-diseased flocks (n=10) were sampled as described in diseased flocks. The collected samples were labeled and transported cooled to the laboratory for processing and bacteriological examination.

### Bacterial isolation

Swabs and internal organs were streaked onto MacConkey agar, sorbitol MacConkey agar (SMA), and incubated aerobically at 37°C for 24 h. Lactose-fermenting colonies (pink colonies) on MacConkey agar and white colonies on SMA were selected and subcultured on brain heart infusion agar for purification. The purified colonies were subjected to Gram staining and oxidase test. Colonies were oxidase-negative, and Gram-negative bacilli were identified biochemically to the species level by the VITEK^®^ 2 COMPACT system using GN identification cards (BioMérieux, France).

### DNA extraction and *16S rRNA* gene amplification and sequencing

Total genomic DNA was extracted from biochemically identified isolates using the QIAamp DNA mini-kit (Qiagen SA, Courtaboeuf, France) according to the manufacturer’s instructions. The *16S rRNA* gene was amplified using the universal primers 27F (5′-AGAGTTTGATCCTGGCTCAG-3′) and 1492R (5′-TACGGYTACCTTGTTACGACTT-3′) according to Weisburg *et al*. [18]. Polymerase chain reaction (PCR) products were purified (QIAquick PCR Purification Kit, Qiagen, France) and sequenced using an ABI 3500 Genetic analyzer (Applied Biosystems, USA). Sequences were subjected to analysis through the National Center for Biological Information (NCBI) Basic Local Alignment Search Tool (https://blast.ncbi.nlm.nih.gov/Blast.cgi).

### Phenotypic detection of ESBL-producing *E. coli* (cephalosporin/clavulanate combination disks)

A standard disk diffusion test was performed according to the Clinical and Laboratory Standards Institute standards and guidelines [19]. Ceftazidime (30 μg) and cefotaxime (CTX 30 μg) disks with or without clavulanate (10 μg) were used for the phenotypic confirmation of the presence of ESBLs in *E. coli*. ESBL production was considered when there was a difference of ≥5 mm between the zone diameters of either of the cephalosporin disks and their respective cephalosporin/clavulanate disks [19].

### Antimicrobial susceptibility

Ten antimicrobials (nine antimicrobial classes), including ampicillin (AMP), amoxicillin-clavulanate (AMC), CTX, cefoxitin (FOX), imipenem (IPM), gentamicin (GEN), ciprofloxacin, tetracycline (TCY), trimethoprim/sulfamethoxazole, and azithromycin, were selected for *E. coli* antimicrobial susceptibility testing. The minimum inhibitory concentration (MIC) for each antimicrobial was determined according to standards and guidelines [19].

### Quantitative detection of biofilm

The microtiter plate (MTP) method described by Naves *et al*. [20] was used to quantify BF. Purified isolates were grown overnight in 15 mL trypticase soy broth (TSB) (Oxoid, UK) supplemented with 1% glucose at 37°C aerobically. Cultures were diluted at 1:100 in freshly prepared sterile TSB. Aliquots of 200 μL from diluted cultures were inoculated in individual wells of sterile flat-bottomed 96-well tissue culture polystyrene plates (Sigma-Aldrich, USA). *E. coli* ATCC 25922 was used as the positive control, and a sterile broth was used as the negative control. Plates were incubated at 37°C for 24 h. After incubation, the broth was removed, and the wells were washed thrice with 200 μL sterile normal saline. After air-drying at room temperature for 20 min, the wells were stained with 200 μL of 1% crystal violet solution for 5 min. After staining, the plates were rinsed with 200 μL sterile deionized distilled water and air-dried for 1 h at 25°C. The optical density (OD) of the stained wells was measured at a wavelength of 540 nm using an enzyme-linked immunosorbent assay reader (BioTek-800 ST, USA). BF was determined according to the formula: BF= AB - CW, where AB is the OD of the wells attached by bacteria, and CW is the OD of the stained control wells. The experiment was performed in duplicate on three different days. Biofilm production by each isolate was scored as either strong (BF=≥0.300), moderate (BF=0.200-0.299), weak (BF=0.100-0.199), or negative (BF=<0.100).

### Detection of virulence genes

Eight virulence genes linked to different *E. coli* pathotypes (*stx1* and *stx2* genes [EHEC], *eae* and *ehxA* genes [EHEC/EPEC], *est* and *elt* genes [ETEC], *bfpA* gene [EPEC], and *aggR* gene [enteroaggregative *E. coli* or EAEC]) and two serotype-related genes (*rbfO157* and *fliCH7* genes [EHEC]) were investigated by real-time quantitative PCR (qPCR) according to the methods of Cabal *et al*. [21]. For the rapid molecular identification of APEC isolates, five specific genes (*iss*, *iutA*, *hlyF*, *ompT*, and *iroN*) were amplified by qPCR according to Ikuta *et al*. [22]. The primers and PCR conditions are illustrated in Table-1 [21,22].

**Table-1:** Primers, probes, and melting temperature used in amplification of different virulence genes.

Primers	Target	Oligonucleotide sequence (5×à 3’)	Melting temperature	Amplicon (bp)	Reference
iss-F	Increases serum survival gene	CGGGAATTGGACAAGAGAAAAC	60	57	[22]
iss-R		TTTCTGCACCGCCACAAA			
FAM		TTTGGCTGCATCAAC			
iutA-F	Ferric aerobactin receptor gene	CGGTGGCGTACGCTATCAGT	60	59	
iutA-R		GCGCGTAGCCGATGAAAT			
VIC		CACTGAAAACAAGATTGAT			
hlyF-F	Putative avian hemolysin	GGTTGCCCGACCATCAATT	60	61	
hlyF-R		ACTGGTTGAAGGTAAGCACCCTAA			
FAM		TTGTTGGCCACAGTCG			
ompT-F	Episomal outer membrane protease gene	GGTTCCGGGATTGCTCGTAT	60	57	
ompT-R		GGTCGTGGAGGCAATATGGT			
VIC		CAGCCAGTCCCTGTC			
iroN-F	Salmochelin siderophore receptor gene	CCGTTGGTGCAGAGTGGAA	60	53	
iroN-R		CAGGCTGGTAGAGGAAGGATCA			
FAM		CGCGATAAGCTCG			
st×1-F	Shiga toxin 1 (st×1)	GCAAAGAMGTATGTWGATTCG	55	107	[21]
st×1-R		GWGCCACTATCAATCATCAG			
ROX		TTCGCTCTGCAATAGGTACKCCAT			
st×2-F	Shiga toxin 2 (st×2)	AATGCAAATCAGTCGTCAC	55	82	
st×2-R		TGCATCTCTGGTCATTGTAT			
FAM		CACTGGTTTCATCATATCTGGCGTT			
eae-F	Intimin	GCTATAACRTCTTCATTGATC	52	92	
eae-R		RCTACTTTTRAAATAGTCTCG			
FAM		TTCGCCACCAATACCTAAACGG			
ehxA-F	Enterohaemolysin (ehxA)	GCACCACAACTTGAYAAACT	55	86	
ehxA-R		CCAGATTATTACCTACATTYTCAG			
FAM		TTTACTCCCAACGTTCTGATACTTCTG			
est-F	ST toxin (est)	TGAAAGCATGAATRGTAGCAA	54	72	
est-R		TTAATAACATSSAGCACAGG			
FAM		CAGGATTACAACAMARTTCACAGCAGT			
elt-F	LT toxin (elt)	GGYAAAAGAGAAATGGTTAT	54	142	
elt-R		TCTCGGTCAGATATGYGATTC			
ROX		TGTGTCCTTCATCCTTTCAATGGC			
bfpA-F	Bundle- forming pilus (bfpA)	CMGGTGTGATGTTTTACTAC	53	109	
bfpA-R		TGCCCAATATACARACCAT			
FAM		AGTCTGCGTCTGATTCCAATAAGKC			
invA-F	Invasion plasmid (spa24)	CCAATCACAATATCAGTACCA	53	159	
invA-R		AAAGAGCCTTATTACCCATAT			
ROX		AGACACATTACCTCCATCATCTAAGCA			
aggR-F	Enteroaggregative regulator (aggR)	TTTATCGCAATCAGATTAARC	56	94	
aggR-R		GGACAACTRCAAGCATCTAC			
ROX		ACATTAAGACGCCTAAAGGATGCC			
rfb O157-F	rbfO157	CAAAAGGAAACTATATTCAGAAGT	55	125	
rfb O157-R		CGATATACCTAACGCTAACAA			
FAM		ATTCCTCTCTTTCCTCTGCGGTC			
wzx O104-F	wzxO104	GCGCAAAGAATTTCAACTT	55	99	
wzx O104-R		TGTAAAATCCTTTAAACTATACG			
ROX		TGAAATGACACCACTTATTGCTAATACA			

### Statistical analysis

The Fisher’s exact test or χ^2^ test and Spearman’s rank correlation test using GraphPad Prism 8 (GraphPad Software, San Diego, CA 92108, USA) were used to determine the statistical significance of the data.

## Results

### Bacterial isolation

**Figure-1:**
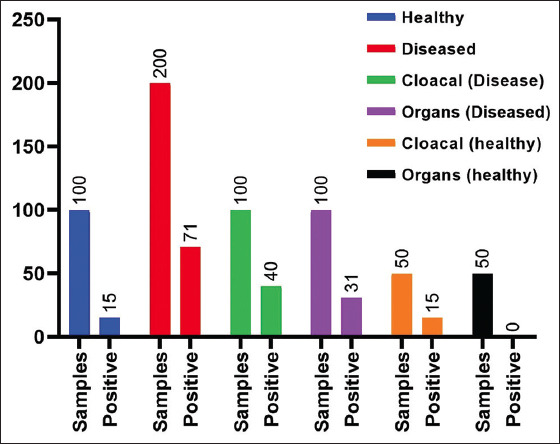
Distribution of *Escherichia coli* isolates (n=86) concerning the clinical status of the flocks and the site of sampling.

Eighty-six *E. coli* strains were isolated from 30 backyard chicken flocks. Isolates were biochemically identified to the species level. Genetically, sequences of the *16S rRNA* gene showed >98% identity with *E. coli* in the NCBI database. Representative sequences were deposited in the NCBI sequences database with GenBank accession numbers MW366906, MW366747, MW368769 to MW368781, and MZ413447 to MZ413460. The frequency of isolation from diseased flocks was significantly higher (p<0.05) than apparently healthy flocks. Figure-1 shows the distribution of isolates concerning the clinical status of the flocks and the sampling site.

### Antimicrobial susceptibility

**Figure-2:**
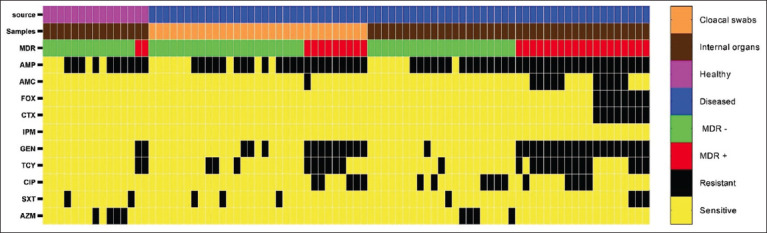
Heat map representation of the antimicrobial-resistant profile of *Escherichia coli* isolates (n=86) recovered from healthy and diseased backyard chickens.

**Figure-3:**
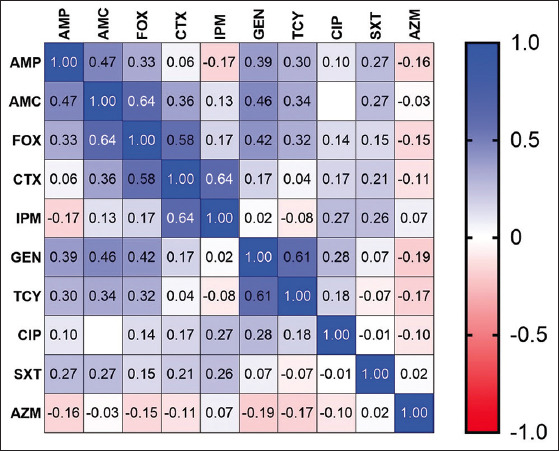
Spearman rank correlation coefficient between the antimicrobials based on minimum inhibitory concentration of 86 *Escherichia coli* isolates.

The antimicrobial susceptibility profile of the 86 *E. coli* isolates is shown in Figure-2. In this study, *E. coli* isolates showed high resistance to AMP (75.6%), GEN (39.5%), and TCY (29.1%). However, none of the isolates were resistant to IPM. The resistance rate was significantly higher (p<0.05) in *E. coli* strains isolated from diseased birds than healthy birds. However, intestinal isolates showed a non-significantly higher (p>0.05) resistance rate than extraintestinal isolates. The MIC50, MIC90, and MIC range for the ten antibiotics are shown in Table-2. The pairwise correlation between antimicrobial MIC values and *E. coli* isolates is shown in Figure-3. The strongest, statistically significant (p<0.001) correlations were between TCY and GEN (r=0.61), FOX and AMC (r=0.64), FOX and CTX (r=0.58), and AMP and AMC (r=0.47). The drug resistance profile of *E. coli* isolates is presented in Table-3. Overall, 21 (24.4%) isolates were sensitive to all ten antimicrobials. Seven (8.1%) isolates were resistant only to AMP, and 28 (32.6%) were resistant to two antimicrobials, whereas the remaining 30 (34.9%) isolates showed multidrug resistance (MDR). Based on the MDR index, 30 (34.9%) isolates showed an MDR index of >0.2, and 28 (32.6%) isolates showed an MDR index of <0.2. Of the 86 isolates, 8 (9.3%) were confirmed as ESBL-producing *E. coli* by the combination disk diffusion method. All ESBL isolates were MDR with an MDR index of 0.5 to 0.6.

**Table-2:** The MIC50, MIC90, and MIC range and resistance percentage of *Escherichia coli* (n=86) isolate from healthy and diseased birds.

Antibiotic	No.	R%	MIC50	MIC90	MIC Range	No. of resistant *E. coli* (%)			

						Clinical status		Sample site	
	
						Normal Birds	Diseased Birds	Cloacal swabs	Internal organs
AMP	65	75.6	64	128	1-164	10 (15.4)	55 (84.6)	43 (66.1)	22 (33.9)
AMC	11	12.8	4	32	1-64	0 (0.0)	11 (100)	10 (90.9)	1 (9.1)
CTX	8	9.3	0.5	1	0.125-16	0 (0.0)	8 (100)	8 (100)	0 (0.0)
FOX	8	9.3	4	8	0.5-64	0 (0.0)	8 (100)	8 (100)	0 (0.0)
IPM	0	0	0.25	1	0.125-1	0 (0.0)	0 (0.0)	0 (0.0)	0 (0.0)
GEN	34	39.5	4	32	1-64	2 (5.9)	32 (94.1)	22 (64.7)	12 (35.3)
CIP	16	18.6	0.125	4	0.125-8	0 (0.0)	16 (100)	11 (68.8)	5 (31.2)
SXT	9	10.5	2	8	1-16	2 (22.2)	7 (77.8)	6 (66.7)	3 (33.3)
AZM	8	9.3	8	16	2-64	4 (50)	4 (50)	8 (100)	0 (0.0)
TCY	25	29.1	4	32	2-64	2 (8)	23 (98)	16 (64)	9 (36)

MDR=Multidrug resistance, AMP=Ampicillin, AMC=Amoxicillin-clavulanate, FOX=Cefoxitin, IPM=Imipenem, GEN=Gentamicin, MIC=Minimum inhibitory concentration, CTX=Cefotaxime, TCY=Tetracycline, SXT=Trimethoprim/sulfamethoxazole, AZM=Azithromycin

**Table-3:** Drug resistance profile and MDR index of *Escherichia coli* isolates (n=86).

Resistance profile	Number of isolates (%)	MAR index
	21 (24.4)	0
AMP	7 (8.1)	0.1
AMP AZM	8 (9.3)	0.2
AMP SXT	6 (7)	0.2
AMP CIP	6 (7)	0.2
AMP TCY	4 (4.7)	0.2
AMP GEN	4 (4.7)	0.2
AMP GEN CIP	4 (4.7)	0.3
AMP GEN TCY	6 (7)	0.3
AMP GEN TCY CIP	6 (7)	0.4
AMP AMC GEN TCY	6 (7)	0.4
AMP AMC FOX CTX GEN	5 (5.8)	0.5
AMP FOX CTX GEN TCY SXT	3 (3.5)	0.6

MDR=Multidrug resistance, AMP=Ampicillin, AMC=Amoxicillin-clavulanate, FOX=Cefoxitin, IPM=Imipenem, GEN=Gentamicin, MAR=Multiple antibiotic resistance, CTX=Cefotaxime, TCY=Tetracycline, SXT=Trimethoprim/sulfamethoxazole, AZM=Azithromycin

### BF

**Figure-4:**
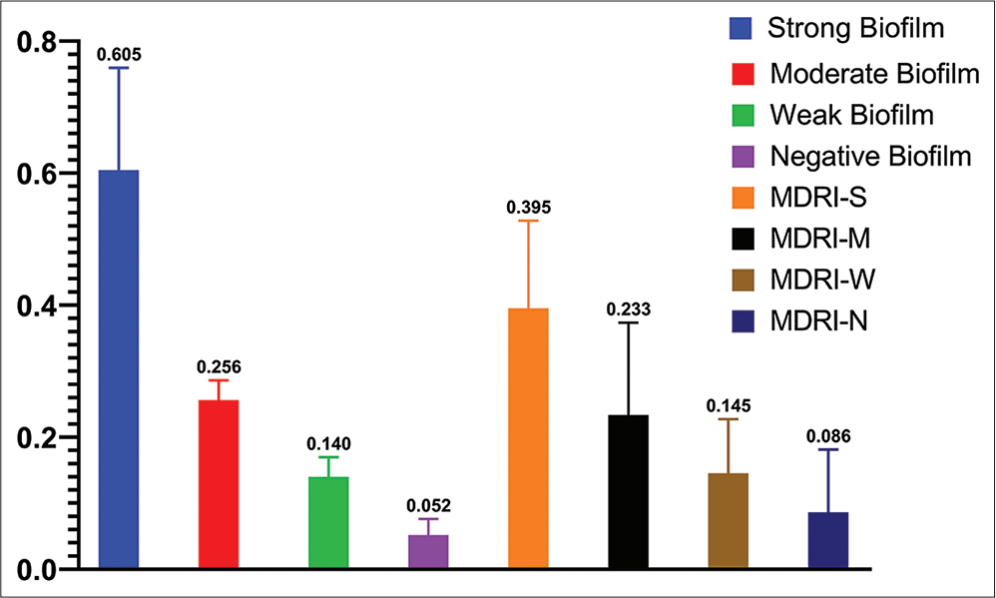
Biofilm formation: the mean OD of strong, moderate, weak, and negative biofilm producer *Escherichia coli* and the mean value of multidrug resistance index in each group.

**Figure-5:**
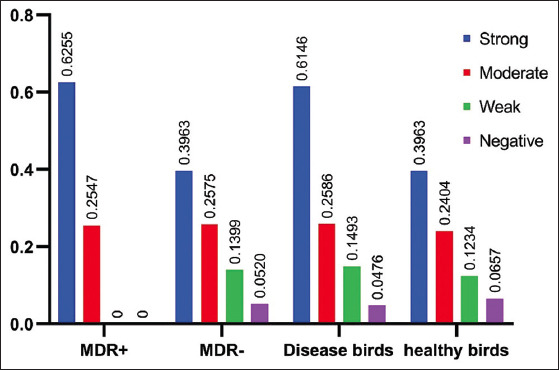
The distribution of the four biofilm formation groups among *Escherichia coli* strains isolated from healthy and diseased birds and the strains that exhibited multidrug resistance profile.

The results of the MTP method revealed that 57 (66.27%) isolates were capable of forming biofilms. Based on the corrected OD_540 nm_ of the bacterial biofilm, the isolates were categorized into four groups: Strong, moderate, weak, and negative. Of the 86 isolates, 22 (25.6%) were strong biofilm producers, 24 (27.9%) moderate producers, 11 (12.8%) weak producers, and 29 (33.7%) were unable to form biofilm. The mean of the corrected OD values for each group is shown in Figure-4. The correlation between both identified MDR isolates and MRD index and BF was investigated. A statistically significant (p<0.001) pairwise correlation was obtained for MDR versus BF (r=0.512) and MRD index versus BF (r=0.556). The mean values of the MDR index in each group are shown in Figure-4. The distribution of the four BF groups among *E. coli* strains isolated from healthy and diseased birds and the strains that exhibited MDR profile is shown in Figure-5. MDR isolates showed a significantly strong and moderate BF than weak and negative producers (p>0.05). *E. coli* strains isolated from diseased birds showed a significant strong BF (p>0.05).

### Virulence genotyping

**Figure-6:**
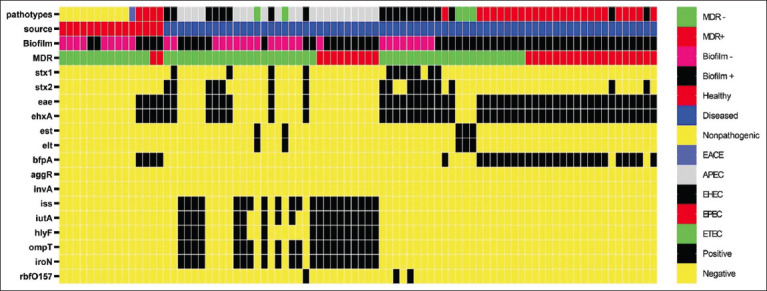
Heat map representation of virulence gene profile of *Escherichia coli* isolates (n=86) recovered from healthy and diseased backyard chickens.

In general, each of the 14 virulence-associated genes was investigated in the 86 isolates. Thirteen virulence genes were identified with varying degrees of frequency; however, the *invA* gene was not identified in any isolate. The higher frequency of virulence genes was statistically significant in *E. coli* isolated from diseased birds than healthy birds (p<0.001). The distribution of different virulence genes is illustrated in Figure-6. Based on virulence gene profiles, five pathotypes were identified: EPEC (39.5%), APEC (32.53%), EHEC (9.3%) EAEC (1.2%), and ETEC (5.8%). The lower frequency of EAEC and ETEC was statistically significant than other pathotypes. Ten (11.6%) isolates were identified as nonpathogenic *E. coli*. A BF was reported in all EPEC pathotypes, and 19 (65.5%) showed MDR profiles. Table-4 shows the virulence genes, BF, and MDR in different *E. coli* pathotypes. Of the eight EHEC pathotypes, three isolates were *stx1*^+^-*stx2*^+^, 2 isolates were *stx1*^+^-*stx2*^-^, and three isolates were *stx1*^-^-*stx2*^+^. Three isolates were identified as O157 based on the detection of the *rbfO157* gene.

**Table-4:** Virulence genes, biofilm formation, and MDR in different *Escherichia coli* pathotypes.

Pathotype	NO	MDR	Biofilm	Virulence genes													

				*stx1*	*stx2*	*eae*	*ehxA*	*est*	*elt*	*bfpA*	*aggR*	*invA*	*iss*	*iutA*	*hlyF*	*ompT*	*iroN*
APEC	28	9	16	0	0	0	0	0	0	0	0	0	28	26	23	28	28
EAEC	1	0	0	0	0	0	0	0	0	0	1	0	0	0	0	0	0
EHEC	8	2	3	5	6	8	8	0	0	0	0	0	0	0	0	0	0
EPEC	34	19	31	0	0	34	34	0	0	34	0	0	0	0	0	0	0
ETEC	5	0	3	0	0	0	0	5	5	0	0	0	0	0	0	0	0
Non-Path	10	0	4	0	0	0	0	0	0	0	0	0	0	0	0	0	0

MDR=Multidrug resistance, EHEC=Enterohemorrhagic *Escherichia coli*, EPEC=Enteropathogenic *Escherichia coli*, ETEC=Enterotoxigenic *Escherichia coli*, APEC=Avian pathogenic *Escherichia coli*

## Discussion

Although backyard chicken flocks have traditionally been regarded as an essential food source in developed countries, backyard chickens may act as a reservoir and spread various zoonotic bacterial pathogens. Free-living chickens in backyard flocks increase the risk of gaining bacterial pathogens from wild animals or birds. Furthermore, close contact with humans facilitates the transmission of zoonotic pathogens [23]. In this study, *E. coli* was isolated from 40% and 31% of cloacal swabs and internal organs, respectively, collected from diseased chickens. However, in healthy chickens, *E. coli* was only recovered from 30% of cloacal swabs. The frequency of isolation in this study was relatively lower than the previously reported study in Malaysia (48%) [24] and similar to that reported in Egypt (34%) [25] and Ethiopia (32.5%) [26]. The variation in isolation frequency may be attributed to the sampling size, type of samples, and breeding system.

Antibiotic resistance development, transmission, and persistence remain major concerns, particularly in low- and middle-income countries, where small-scale animal husbandry is prevalent [27,28]. In this study, *E. coli* isolates showed variable resistance to the ten antibiotics. High resistance was reported to AMP (75.6%), GEN (39.5%), and TYC (29.1%). This result was consistent with previous studies [29-31], which reported 74%, 41%, and 32% resistance to the three antibiotics in Brazil, Jordan, and Thailand, respectively. However, higher resistance to AMP (94%) and TYC (100%) was reported in Zimbabwe [32] and Spain [33], whereas a lower resistance to GEN (24%) was reported in Thailand [31].

The results revealed that 34.9% of the isolates presented MDR profiles to three and up to six antimicrobials. The emergence of MDR *E. coli* has been previously reported and is currently regarded as a growing public health concern [34-36]. The high incidence of antimicrobial resistance reported within or between antimicrobial classes in multiple investigations might be attributed to the extensive, indiscriminate, and long-term usage of comparable medicines in chicken farms [37].

In this study, 9.3% of the isolates showed ESBL activity; however, carbapenemase activity was not observed in any isolate. These findings were corroborated by earlier studies on the presence of ESBL-producing *E. coli* in chickens [34,38,39]. However, a higher frequency of ESBL (58.6% and 29%) was reported in Egypt [40] and Ghana [41], whereas a lower frequency (5.1%) was recorded in Germany [42]. ESBL activity in Enterobacteriaceae poses a severe public health risk because of the ability of these bacteria to hydrolyze third-generation cephalosporins classified by the World Health Organization as the highest priority critically important antimicrobials for human medicine [43].

BF by *E. coli* has been recognized as an essential factor associated with its virulence [44]. In this work, BF was detected by the MTP method in 66.27% of the isolates. Similar results (62.5% and 68%) were reported in Uganda [11] and Czech Republic [45], and relatively lower (55.8%) BF was reported by Rodrigues *et al*. [12]. *E. coli* recovered from diseased birds showed a significantly strong BF. These findings were confirmed by Lewis [46], who reported that bacterial biofilms are considered to be involved in ~65% of all bacterial infections. This study revealed a statistically significant correlation between MDR and BF. This agreed with previous reports in Uganda [11] and Spain [47]. High resistance of biofilm bacteria to antimicrobials is a critical issue in the treatment of infections, and biofilm cells are thought to be 100-1000 times more resistant to antimicrobial treatments than planktonic bacterial cells [10,48].

qPCR is a rapid and accurate method for diagnosis. In this study, 14 virulence genes were detected by TaqMan real-time PCR, and 24.4% of the isolates were identified as APEC based on the five common genes (*iss*, *iutA*, *ompT*, *hlyF*, and *iroN*). A relatively higher frequency (36.36%) was reported in Bangladesh [49] and a lower frequency in Brazil [50]. APEC is the causative agent of colibacillosis, a highly fatal septicemic disease of chickens that causes significant economic losses in the poultry industry worldwide [51]. Unfortunately, eight isolates were identified as EHEC pathotypes. This result was consistent with Wang *et al*. [52] and documented by Kim *et al*. [5], who reviewed that domestic poultry, including turkeys, duck, and chicken, are reservoirs for EHEC.

## Conclusion

This study investigated the antimicrobial resistance, virulence genes, and BF in *E. coli* strains isolated from backyard chicken in the eastern region of Saudi Arabia. High resistance to different classes of antimicrobials was identified, suggesting the misuse of antimicrobials in backyard chicken farms. The emergence of ESBL and EHEC isolates in backyard chickens is a public health concern. The environment of backyard flocks may harbor different pathogenic bacteria that may enhance the persistence of infection and the transmission to in-contact humans. Therefore, regular monitoring for the occurrence of MDR and the zoonotic pathotypes among *E. coli* in backyard chicken flocks is recommended, as these bacteria can transmit to humans through food products or contaminated environments.

## Authors’ Contributions

MF and TA: Conceptualization; MF, TA, AA, RA, and AAA: Methodology. MF and RA: Software. MF and AAA: Validation. TA and AA: Formal analysis. AA, AAA, and RA: Investigation. TA and RA: Resources. AA, AAA, and RA: Data curation. MF and TA: Original draft preparation. MF, TA, and RA: Review and editing. TA, AA, and AAA: Visualization. MF: Supervision. All authors read and approved the final manuscript.
